# Blueberry as an Attractive Functional Fruit to Prevent (Pre)Diabetes Progression

**DOI:** 10.3390/antiox10081162

**Published:** 2021-07-22

**Authors:** Sara Nunes, Pedro Vieira, Pedro Gomes, Sofia Domingues Viana, Flávio Reis

**Affiliations:** 1 Institute of Pharmacology & Experimental Therapeutics & Coimbra Institute for Clinical and Biomedical Research (iCBR), Faculty of Medicine, University of Coimbra, 3000-548 Coimbra, Portugal; spnunes@fmed.uc.pt (S.N.); pedromdvieira96@gmail.com (P.V.); pgomes70@gmail.com (P.G.); 2 Center for Innovative Biomedicine and Biotechnology (CIBB), University of Coimbra, 3004-504 Coimbra, Portugal; 3 Clinical Academic Center of Coimbra (CACC), 3004-504 Coimbra, Portugal; 4 Department of Biomedicine, Faculty of Medicine, University of Porto, 4200-450 Porto, Portugal; 5 CINTESIS—Center for Health Technology and Services Research, University of Porto, 4200-450 Porto, Portugal; 6 Pharmacy/Biomedical Laboratory Sciences, Polytechnic Institute of Coimbra, ESTESC-Coimbra Health School, 3046-854 Coimbra, Portugal

**Keywords:** blueberries, antioxidants, prediabetes, hepatic dysmetabolism, gut microbiota dysbiosis

## Abstract

Prediabetes, a subclinical impairment between euglycemia and hyperglycemia, is a risk factor for the development of type 2 diabetes mellitus (T2DM) and associated micro- and macrovascular complications. Lifestyle therapy, the first-line treatment of prediabetes, includes physical exercise and dietary regimens enriched in phytochemicals with health-related properties. Blueberries (*Vaccinium* spp.), given their pleasant taste and great abundance in beneficial phytochemicals, have gained public interest all over the world. Along with a high antioxidant activity, this functional fruit is also well-recognized due to its hypoglycemic and insulin-sensitizing effects and has been recommended for overt T2DM management. Yet blueberries target several other pathophysiological traits, namely gut microbiota dysbiosis and hepatic dysmetabolism, that ensue when prediabetes begins and for which pharmacological interventions tend to be delayed. In this work, we revisited preclinical data from in vitro assays, animal models and human studies, aiming to disclose the potential mechanisms by which blueberries may be a fruitful source of phytochemicals able to prevent (pre)diabetes progression. Collectively, future efforts should focus on longer-term studies with standardized interventions and readouts, particularly in humans, that will hopefully bring more robust evidence and concrete guidance for blueberries’ effective use in prediabetes.

## 1. Introduction

The type 2 diabetes mellitus (T2DM) epidemic is a major public health concern [1]. According to the International Diabetes Federation (IDF) estimates, 463 millions of people are living with diabetes globally, and it is projected to rise to 700 million by 2045 [2,3]. Paralleling this daunting scenario, prediabetes (also referred as intermediate hyperglycemia by the World Health Organization (WHO) [4] is also an increasingly common condition, characterized by blood glucose levels above the normal range but below diabetes diagnostic thresholds, and typically defined as a state of impaired fasting glucose (IFG) and/or impaired glucose tolerance (IGT). While the IDF has no data about the prevalence of IFG, 373 million people worldwide are estimated to have IGT, a number that is predicted to increase to 548 million by 2045 [2,3].

Although the prevalence of prediabetes varies according to the criteria used for diagnosis (WHO vs. American Diabetes Association, ADA) and the study population, it is consensually recognized as a risk state for T2DM development [5]. In fact, both insulin resistance and impaired β-cell function, which are disease hallmarks and early pathophysiological mechanisms, are responsible for the increased risk of T2DM development in patients with prediabetes [6]. Evidence points to a yearly conversion rate of 5–10% [5,7,8], but also to cardiovascular disease (CVD) and even cancer [9,10]. Multiple genetic and environmental risk factors contribute to the progression of the disease, namely the age of the population, its genetic background, a sedentary lifestyle and poor (hypercaloric) nutritional habits [5,11], collectively leading to obesity, hepatic steatosis and dyslipidemia, which are important predisposing factors for the progression of T2DM and CVD [12].

Along with peripheral insulin resistance and defects in insulin secretion, other unbalanced metabolic processes, including glucotoxicity and lipotoxicity, oxidative stress and low-grade inflammation are already evolving in the state of prediabetes [6]. In addition, recent evidence suggests that changes in gut microbiota (GM) community may also play a role in T2DM pathophysiology and impaired composition and diversity of intestinal bacterial flora (referred as dysbiosis) may actually begin at an early stage [13,14].

Lifestyle therapy, including regular physical activity and diabetes-specific nutrition therapy, is the cornerstone for the early management of prediabetes due to their accessibility, cost-effectiveness as well as the limited pharmacological options with favorable safety profiles namely in the earlier stages of the disease [15,16,17,18]. Accordingly, prospective and meta-analyses studies revealed an inverse correlation between fruit and vegetable intake and the increased risk and incidence of T2DM [19,20,21]. Moreover, the potential beneficial roles of phytochemicals (e.g., polyphenols, prebiotic fibers) and macronutrients in plant-based diets for the prevention of metabolic syndrome and T2DM are emerging [22,23,24]. Among various bioactive components, epidemiological studies and randomized clinical trials clearly demonstrate that diets with high-content of polyphenolic compounds, high antioxidant capacity and prebiotic/probiotic/symbiotic activities are inversely associated with the risk of insulin resistance and (pre)diabetes progression [25,26,27,28,29]. Blueberries (BB), due to their enriched content in the mentioned phytochemicals, have enticed keen interest as a feasible dietary intervention for the prevention and management of (pre)diabetes. Notably, both observational and clinical studies as well as cumulative preclinical data strongly underscore BB as an attractive complementary strategy to halt the progression from prediabetes to overt T2DM [20,30,31]. Besides their well-recognized hypoglycemic and insulin-sensitizing effects [32], BB-enriched diets further improve GM homeostasis [33,34,35] and lessen hepatic dysmetabolism [36], two key components of (pre)diabetes evolution.

In this work, we review current evidence linking the benefits of consuming BB with hepatic- and gut-derived pathophysiological traits ensuing as soon as prediabetes. Moreover, we provide an updated analysis of the main cellular and molecular mechanisms by which BB phytochemicals are able to exert protective effects.

Studies search was performed in PubMed and ClinicalTrials.gov databases using a strategy based on keywords. Most consistent, relevant and well-designed studies (both original and literature reviews) published between 2000–2021 were included. Collectively, this work scrutinizes forefront evidence in support of BB-enriched diets as an attractive option to prevent (pre)diabetes progression.

## 2. From Prediabetes to Overt Diabetes—Emphasis on Liver and Gut Paths

The transition from a normal state to prediabetes, and then to diabetes, is the consequence of a series of pathophysiological changes, that makes the individual gradually more susceptible to the subsequent disruption of glucose homeostasis, which is mainly governed by insulin sensitivity of peripheral tissues and pancreatic β-cell function. Although there has been a significant debate in the past on whether insulin resistance or β-cell dysfunction is the primary driver in the pathogenesis of prediabetes as well as on their relative contributions to disease development [37], the current view is that both conditions dynamically influence each other and presumably synergistically exacerbate diabetes [38]. Increased peripheral insulin resistance and altered β-cell function leads to major biochemical alterations in metabolic tissues, including the liver, gut, skeletal muscle and adipose tissue, among other organs. Notably, oxidative stress and low-grade chronic inflammation seem to be major molecular mechanisms underlying (pre)diabetes progression, both as a cause or consequence of hyperglycemia and hyperinsulinemia [39,40,41].

In addition to being the main detoxifying organ of the body, the liver also plays a fundamental role in maintaining energy and metabolic homeostasis. Disturbances in hepatic glucose and lipid metabolism similarly contribute to the development of metabolic diseases, including T2DM and non-alcoholic fatty liver disease (NAFLD) [42]. Because glucose is a readily available energy source for cells, the maintenance of blood glucose levels within a relatively narrow range during periods of nutrient shortage or excess is critical for survival. The liver plays a major role in maintaining whole body glucose homeostasis by regulating several metabolic processes involved in glucose transport, oxidation, storage and production. Simultaneously, hepatic lipid metabolism encompasses fatty acid uptake, synthesis, oxidation, storage, and secretion, which requires a well-orchestrated balance between these processes, dietary intake and energy expenditure [43]. Hepatic steatosis—the hallmark and earliest feature of NAFLD—is defined as an excessive accumulation of TGs in the cytoplasm of hepatocytes due to imbalances in the processes that maintain the normal hepatic fatty acid metabolism, and often occurs in the setting of overnutrition and obesity [44,45]. Collectively, the data available support the notion that hepatic steatosis is a favorable biological milieu for the development and progression of (pre)diabetes [46]. Several potential mechanisms could explain the association between hepatic steatosis and the onset of prediabetes, including increased levels of hepatokines and the presence of hepatic insulin resistance [47,48].

Hundreds of trillions of microorganisms inhabit the human body, the overwhelming majority bacteria from two main phyla (Bacteroidetes and Firmicutes). This complex bacterial community is present in many body fluids and surfaces but has a huge implantation in the gastrointestinal tract (GIT), in particular in the large intestine. GM can be seen, in a way, as a supra-organism that performs crucial functions for host homeostasis (co-metabolism), which include metabolic and energetic effects, protection against pathogens, maintenance of intestinal barrier integrity and education of immune system [49,50]. Disturbances in the composition and/or functions of GM cause a situation referred as dysbiosis which has been associated not only with disorders of the GIT, but also with extra-intestinal diseases, including metabolic disorders such as obesity and diabetes [51,52]. Accumulating evidence indicates that GM is involved in host metabolism by increased energy extraction, immune system modulation, and altered lipid and glucose metabolism, all which have been demonstrated to contribute to progression to T2DM. It has been also shown that an individual’s glucose response after a meal is influenced by a combination of their GM composition and host physiology [53,54]. Several studies have reported decline of butyrate-producing bacteria in prediabetes cohorts of distinct ethnicities [55,56,57,58], and to a greater extent in people with both IGT and IFG [13]. In sum, these findings suggest causal links between microbiota dysbiosis and glucose intolerance/progression to T2DM.

## 3. Blueberries Beneficial Outcomes in (Pre)Diabetes Progression—A Multi-Modal Functional Fruit

### 3.1. BB Phytochemistry, Metabolism and Bioavailability—An Overview

Blueberry is one of the five healthy fruits recommended by the Food and Agriculture Organization from United Nations [59]. It belongs to the genus Vaccinium of Ericaceae family which includes approximately 450 species all over the world [60]. Some examples are the well-known *Vaccinium corymbosum*, *Vaccinium ahei*, *Vaccinium poasanum, Vaccinium angustifolium* and *Vaccinium myrtillus*, the later commonly denominated as bilberries across Europe [61,62]. BB are characterized by their enriched nutritive composition, containing high contents of a wide diversity of biologically active components. BB chemical composition greatly varies upon the cultivar, variety, growing location, environmental conditions, plant nutrition, ripeness stage, harvest time as well as the storage conditions, making the content of each individual component highly flexible, with impact on the corresponding antioxidant profiles [63,64,65]. Nonetheless, BB-derived phytochemicals display a panoply of health-related properties beyond their antioxidant profile able to interfere with chief physiological functions [66,67,68]. Therefore, public interest in BB as a functional food has increased over the last years [69]. Even though the aerial parts of the shrubs are used as a folk medicine for treating metabolic diseases for years, the fruits have been constantly increasing in popularity due to their pleasant taste and organoleptic acceptance as well as the richness of their bioactive compounds [70].

BB fruits are low in calories (0.046 kcal/g fresh fruit) and high in water, micronutrients (e.g., selenium, zinc, iron), dietary prebiotic fibers (3–3.5% of fruit weight), vitamins (C, B complex, E, and A) and sugar units [e.g., glucose (≈26.415 mg/g–46.495 mg/g fruit), fructose (≈22.682 mg/g–43.074 mg/g/fruit) and other osidic moieties such as ribose, rhamnose, arabinose and maltose] which are often associated with bioactive polyphenols (PP), known as heterosydic forms of PP [60,71,72,73]. Briefly, PP are secondary metabolites of the plants that act against ultraviolet radiation as well as in plant-microbe interactions and defense response [74]. Collectively, they encompass an aromatic ring and a minimum of two hydroxyl substituents which impact ROS scavenging ability and subsequent antioxidant effects depending on their number and position [75]. PP comprise a large family of compounds and are classified as flavonoids (including flavonols, flavanols, flavones, isoflavones, anthocyanidins and flavanones), phenolic acids (including benzoic acids, cinnamic acids and derivatives), chalcones and coumarins (intermediate in the biosynthesis of flavonoids), and lastly, polymers, including tannins and lignin [76]. PP are present in distinct parts of the BB fruit; for instance, while tannins and phenolic acids are mostly predominant in the seeds, anthocyanins and proanthocyanidins are present mainly in the fruit’s skin and pulp, respectively [77]. Moreover, PP occur in vegetable cells in soluble forms (mostly localized in the cell vacuoles) as well as covalently bound with cell wall macromolecules such as structural proteins, cellulose, hemicellulose, arabinoxylans and/or pectins, collectively designated as PP insoluble forms [78]. Likewise, insoluble PP naturally present in BB fruits may become tightly linked (e.g., esther; C-C bonds) with pectin and xyloglucan, two main components of BB cell wall with prebiotic activity [35].

The biological properties of PP found in BB fruits greatly depend on effective concentrations reached in internal compartments that need to be maintained for an adequate period of time [79]. Notably, the poor bioavailability of PP present in ingested food, ranging from 1–5% of the total PP intake [80,81,82], is inversely correlated with their biological effects, a phenomenon stated as the low bioavailability/high bioactivity paradox [83]. Even though PP are extensively metabolized within enterocytes, colonic GM and liver by phase I/II enzymatic reactions (e.g., sulfation, methylation, glucuronidation, PP ring-fission), a wide variety of new chemical structures retaining intrinsic bioactivity along with parent compounds reach systemic circulation and are distributed to different organs and tissues, supplying efficient cellular concentrations that underpin PP efficacy [82,83,84].

PP physicochemical properties, their release from the food matrix during GIT digestion (bioaccessibility), cellular uptake, metabolism and transport in the circulatory system are key features that determine PP organism availability [80]. To better understand blueberry PP metabolism and pharmacokinetics, Zhong and colleagues carried out a single blind, randomized trial and followed human plasma concentrations of anthocyanins, chlorogenic acid and their metabolites over 24 h after oral ingestion of a wild blueberry beverage. The total bioavailability of unaltered anthocyanin compounds (displaying their original C6-C3-C6 structure) and chlorogenic acid was 1.1% and 0.2%, respectively. Parent compounds and metabolites (e.g., cyanidin-, delphinidin-, petundin-glucoronide metabolites) peaked in plasma within the first 1–4 h post-ingestion (early phase response) and >5 h after ingestion (late phase response). This bi-phasic response, probably reflective of enterohepatic circulation, may imply that different BB-derived PP and metabolites interact with GIT cells and other tissues at different times post-consumption [85]. As a matter of fact, nanostructures comprising PP-BB matrix conjugates may be protected along the transit through the GIT, a phenomenon affecting both PP absorption and cellular uptake. Likewise, in vitro gastrointestinal digestion of BB fruits underscored a high stability of total PP and anthocyanins during the gastric digestion step along with decreased contents upon intestinal breakdown (49% and 15%, respectively). This profile paralleled unchanged antioxidant activity following gastric digestion and a significant reduction (<50%) upon digestion under intestinal conditions [80].

Furthermore, it is generally accepted that the remaining unabsorbed PP glycosides and conjugates cycled through hepatic and ileal metabolism reach the colon and modulate fermentation towards a myriad of health benefits [83]. Since small intestine lacks pectinases and cellulases, PP-dietary fibers conjugates are disrupted by colonic GM enzymes and PP metabolites liberation occurs [78,86]. Such metabolites display chief functions in colon health, modifying gut microbial balance towards beneficial bacteria prevalence and SCFAs production [87,88]. In vitro colonic digestion has also shown BB polyphenols biotransformation to syringic, cinnamic, caffeic and protocatechuic acids [80]. Likewise, microbial metabolism of blueberry PP was found to elicit positive outcomes, namely the inhibition of HT-29 colon cancer cell line proliferation as well as the repression of colonic prostanoid production and anti-inflammatory effects [89,90]. Overall, the in vivo bioactivity of blueberry polyphenols is strictly correlated with their absorption along the GIT as well colonic microbial metabolism [80].

### 3.2. Hypoglycemic and Insulin Sensitizing Effects of BB

A number of in vitro studies strongly suggests that BB and its bioactive phytochemicals present anti-diabetic properties. The ability of an extract from Lowbush blueberry fruit to increase pancreatic β-cell proliferation was explored in a cell culture-based bioassay, suggesting a potential capacity to restrain β-cell damage and improve insulin sensitivity [70]. Likewise, whole BB afforded pancreatic β-cell protection and prevented β-cell apoptosis and expansion in HFD-fed C57BL/6J mice [91]. Notably, a tight regulation of pancreatic islet area was observed as blueberry supplementation induced an increased number of small islets (comprising more β-cells with higher insulin) along with a decreased density of larger islets, probably delaying the overwhelmed burden of β-cells activity that pair obesity and diabetes progression [91,92]. Similar pro-proliferative effect was observed for a BB leaf extract in pancreatic MIN6 β-cells, accompanied by improved insulin signaling [68]. In vivo, this extract was able to decrease BW, plasma glucose, HbA1c, HOMA-IR, TGs and NEFAs levels in C57BL/6J mice fed with HFD [68]. These effects paralleled an increased expression of pancreatic β-cell proliferation-related genes (Ngn3, MafA, Pax4, Ins1, and Ins2) and insulin signaling genes (IRS-1, IRS-2, PIK3ca, PDK1, PKCε, and GLUT-2) while FoxO1, a β-cell apoptosis-related gene, was found downregulated [68]. Similar results were observed in male Wistar rats submitted to a combined STZ+HFD paradigm of experimental T2DM [93].

**Figure 1:**
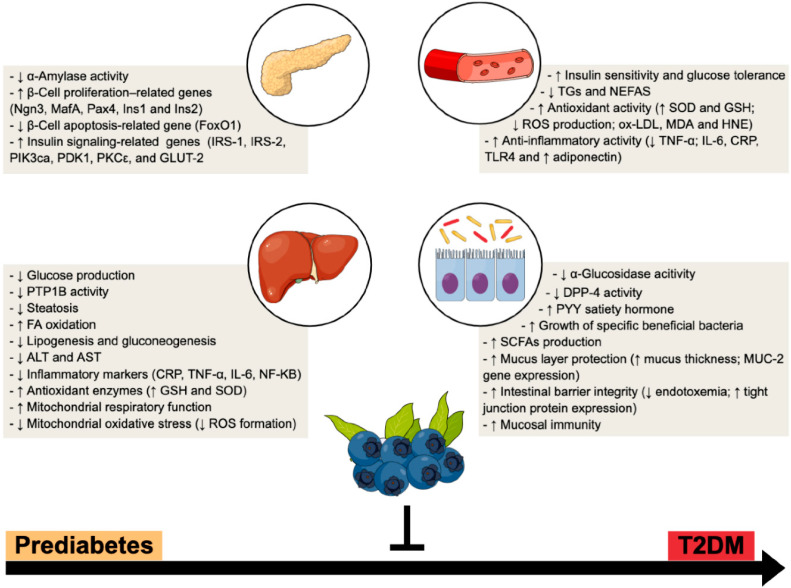
BB multi-organ impact within T2DM progression—a multi-modal functional fruit. Preclinical and clinical studies suggest a broad range of systemic and tissue (e.g., pancreas, liver and gut) beneficial mechanisms, which reinforces BB as an attractive nutraceutical to counteract the progression from prediabetes to overt T2DM. ↑, increased; ↓, decreased.

Furthermore, it has been reported that BB and their bioactive compounds exhibit inhibitory activity toward pancreatic α-amylase and intestinal α-glucosidase, two enzymes involved in the metabolism of starch, which could lead to a delay of carbohydrate digestion and/or decreased glucose absorption [94,95,96]. Interestingly, in vitro studies have also highlighted that polyphenols from BB can act as strong DPP-4 inhibitors, analogous to clinically approved pharmacological options (e.g., gliptins) [97,98,99] (Figure 1).

Protein tyrosine phosphatase 1B (PTP1B) is another promising pharmacological target for the treatment of T2DM as it can reduce blood glucose levels by increasing insulin sensitivity [100]. Notably, Tian et al. [101] identified anthocyanins isolated from BB as selective inhibitors of PTP1B able to increase glucose consumption on HepG2 (human hepatoma) cells in a dose-dependent manner. Moreover, Nachar et al. [102] performed an in vitro study with phenolic compounds from BB juice fermented with *Serratia vaccinii* and observed a strong antidiabetic potential in the liver and skeletal muscle, as it was able to regulate key enzymes involved in glycogen synthesis, gluconeogenesis and skeletal muscle glucose uptake.

In vivo pre-clinical and clinical studies also hint for T2DM improvements upon BB consumption. Regarding animal models, phenolic compounds from a fermented BB–blackberry beverage were able to attenuate fasting blood glucose and the development of obesity in C57BL/6J mice [67]. Vendrame et al. [103] also reported that wild BB consumption significantly decreased plasmatic concentrations of HbA1C, resistin, and RBP4 in the obese Zucker rat model of metabolic syndrome along with the repression of hepatic resistin and RBP4 in the abdominal adipose tissue.

Some clinical trials aimed to analyze the potential anti-diabetic effects have also been explored [31,104] (Table 1). In T2DM male volunteers, a single oral capsule of 0.47 g standardized bilberry extract (36% *w*/*w* anthocyanins), equivalent to 50 g of fresh bilberries, significantly decreased the area under the curve (AUC) for both glucose and insulin in 18%, with no changes on gut incretin hormones (GIP and GLP-1) nor in glucagon and amylin secreted from the pancreas [104]. Moreover, an intervention study showed that daily consumption of a BB smoothie (45 g of blueberry bioactive, equivalent to ~2 cups of fresh BB per day) for 6 weeks significantly improved insulin sensitivity in thirty-two obese, insulin resistant adults, despite unchanged fasting serum glucose levels [31]. Nevertheless, recent clinical trials reported inconsistencies on the efficacy of BB in ameliorating glucose profile in both T2DM and insulin resistant subjects [30,105,106]. A double-blind, randomized controlled trial (RCT) was carried out to evaluate the effect of BB consumption on cardiometabolic parameters in type 2 diabetic men [105]. Twenty-six participants consuming a beverage made from 22 g of freeze-dried BB (equivalent to 1 cup of fresh BB) daily for 8 weeks had lower HbA1c and fructosamine levels (two biomarkers of glycemic control) along with lower triglyceridaemia when compared to participants consuming the placebo. However, no significant changes in total body weight, blood pressure, lipid profile (total cholesterol, LDL and HDL-cholesterol) as well as in serum fasting glucose/insulin concentrations were detected. All of these participants were taking noninsulin diabetes medication and had a good glycemic control [105]. In another study based on a long-term RCT, thirty-seven adults with metabolic syndrome received for 6 months a single-serve sachets with 26 g of freeze-dried BB (1 cup of BB, equivalent to 150 g of fresh BB) daily. Improved markers of vascular function and elements of lipid status (increased HDL-cholesterol and alipoprotein A-I) were detected following 1 cup of blueberries daily for 6 months. However, no benefits were observed on insulin resistance and sensitivity, measured by HOMA-IR, quantitative insulin sensitivity index (QUICKI) and HbA1c and by the gold standard 2-step, hyperinsulinemic euglyceminc clamp [107]. This ambiguity between human intervention studies may be due to the heterogeneity of study population, the duration of the study and/or with the specific analytical technique used to assess the glycemic control. In addition, variations in sources and doses of BB-derived phytochemicals, distinct food or beverage matrix or processing may be contributing to the inconsistent findings when assessing the effects on metabolic outcomes.

Notably, it is also challenging to identify the optimal dose of polyphenols needed to improve metabolic health since the amount of bioactive compound present in the aforesaid BB paradigms greatly varies between published studies.

### 3.3. Antioxidant and Anti-Inflammatory Properties of BB

BB owe their antioxidant activities mainly to their phenolic compounds including phenolic acids, condensed tannins and principally, anthocyanins [117,118]. Several studies point to linear correlation between antioxidant activity and the total phenolic concentrations as well as anthocyanins in BB [119,120,121]. Besides anthocyanins, cinnamic acid derivatives of phenolic acids (e.g., caffeic and chlorogenic acid) were found to be more active antioxidants than benzoic acids derivatives [118].

Chronic hyperglycemia leads to a prooxidant and inflammatory scenario. Conversely, increased oxidative stress and inflammation foster insulin resistance and impaired insulin secretion. Hence, treatments aimed to inhibit ROS overproduction and inflammatory markers are welcome to delay the onset and progression of T2DM and related complications [122].

Health beneficial effects of BB phytochemicals are well recognized on the basis of their potent antioxidant and anti-inflammatory properties, particularly in metabolic conditions such as T2DM [96,123,124] (Figure 1). In diabetic human aortic endothelial cells, BB phytochemicals were able to restore cell surface glycosaminoglycan, attenuate endothelial inflammation, suppress monocyte binding and reduce endothelial IL-8 and vascular cell adhesion molecule 1 (VCAM-1) [125]. Additionally, BB anthocyanins extract (BAE) was able to decrease ROS generation while increasing catalase and superoxide dismutase (SOD) activities in high glucose-induced injury of human retinal capillary endothelial cells (HRCECs); furthermore, BAE was also found to influence angiogenesis by decreasing *vascular endothelial growth factor* (VEGF), intercellular adhesion molecule-1 (ICAM-1) and Akt pathways inhibition [123].

Several in vivo studies highlighted the antioxidant and anti-inflammatory effects of BB and its specific compounds on metabolic impairments. An overall improvement in the inflammatory status was reported in obese Zucker rats supplemented with wild BB powder [126]. The consumption of 8% wild blueberry- enriched diet for 8 weeks significantly decreased plasma levels of TNF-α, IL-6 and CRP and increased adiponectin. In addition, the expression of CRP was reduced in the liver, while TNF-α, IL-6 and NF-κB were down-regulated in both liver and abdominal adipose tissue [126]. In male Wistar rats, BB supplementation in the diet elicited the normalization of TNF-α and IL-1β levels, which were increased by 300% and 500% due to a high-fat feeding paradigm [127]. Other authors found that BB powder afforded protection against inflammation of adipose tissue by preventing the upregulation of inflammatory genes (i.e., TNF-α and IL-10) and attenuating oxidative stress (with an increased glutathione peroxidase gene expression), collectively ameliorating obesity-induced insulin resistance [128]. In addition, obese Zucker rats treated during 15 weeks with BB showed improved glucose tolerance and renal function, together with amelioration of oxidative balance (upholding SOD/catalase antioxidant levels while attenuating oxygen and nitrogen free radical production) and inflammatory pathways (decreased gene and protein expression of TLR4, phosphorylation of ERK and p38-MAPK, NF-kB activity) [129]. Moreover, other studies showed an antioxidant effect of bilberry leaves extract in serum of male Wistar rats under a double challenge composed of HFD and STZ [93]. Likewise, in a recent study from our group, long-term supplementation of BB juice boosted serum antioxidant activity in a healthy Wistar rats [130]. Nevertheless, there has been some controversy on BB-derived polyphenols benefits on total antioxidant activity in both in vivo and human studies [131,132,133,134,135]. Distinct BB doses, food matrices and/or alternative methodologies to assess total antioxidant status may foster such ambiguity.

In humans, previous studies indicated that BB can have immunomodulatory effects and reduce oxidative stress in adults with metabolic syndrome [109]. However, the results so far are relatively inconclusive, possibly due to considerable inter-individual variation. In a 8-week RCT, Basu et al. found that circulating levels of oxidized LDL, MDA and hydroxynonenal (HNE) decreased in participants with metabolic syndrome that consumed 50 g of freeze-dried BB beverage (approximately 350 g fresh BB) daily, despite no significant changes in serum glucose levels, lipid profiles or inflammation biomarkers [110]. In another RCT designed to evaluate the effects of 6-weeks intake of 45 g of freeze-dried BB per day in subjects with metabolic syndrome, a significant decrease in superoxide and total ROS contents were found in whole blood and monocytes, together with decreased circulatory inflammatory markers and reduced gene expression of TNFα, TLR4 and IL-6 in monocytes [109]. Similarly, other researchers have found that BB reduced inflammatory markers as well as enhanced antioxidant activity in individuals with metabolic syndrome or diabetes [109,111].

### 3.4. Hepatoprotective Effects of BB

Apart from high antioxidant and anti-inflammatory properties, BB may exert antidiabetic effects by additional mechanisms. It has been unveiled that BB phytochemicals can provide beneficial effects in T2DM by interfering with energy homeostasis and nutrient metabolism in different tissues, including in the liver [107,136].

Several phytochemicals abundant in BB, such as anthocyanins, have been shown to display lipid-lowering actions through hepatic metabolism pathways, including the regulation of key hepatic enzymes involved in lipogenesis and fatty acid catabolism [66,137], as well as in carbohydrate metabolism [94]. A previous in vitro study suggested that concentrate anthocyanins and other polyphenols from BB may improve glucose metabolism by repressing glucose production in H4IIE rat hepatocytes [24]. Additionally, two BB methanolic extracts showed hypoglycemic effects in human non-tumor hepatic LO2 cells, an effect correlated with the increase in GLUT-2 and PPAR-γ expression as well as with the inhibition of relevant inflammatory pathways [59].

The hepatoprotective effects of the BB phytochemicals have been highlighted in several animal models of chemical-induced liver injury [36,138,139] but also in obesity and diabetes-associated chronic diseases, including in NAFLD [36,136,140,141,142], as summarized in Table 2. A recent study showed that nonacylated anthocyanins from bilberry reduced plasma glucose, lipids and BCAA level in obese diabetic ZDF rats, suggesting an improvement of insulin sensitivity and reduction in lipogenesis [143]. In another study in diabetic mice, antidiabetic effects of dietary anthocyanin-rich bilberry extract (BBE) were associated with targeting of hepatic AMPK, GLUT-4 and metabolic enzymes; furthermore, BBE downregulated the mRNA expression of two gluconeogenic enzymes, phoshoenolpyruvate carboxykinase (PEPCK) and glucose-6-phosphatase (G6Pase), and suppressed glucose flux into the blood [136]. In addition, an enhanced phosphorylation of acetyl-CoA carboxylase (ACC), the key enzyme for FA synthesis that is downstream of AMPK, and the upregulation of PPAR-α, acyl-CoA oxidase (ACO) and CPT1, were found in the liver of diabetic mice supplemented with BBE. Furthermore, supplementation of BB polyphenols extracts also decreased the hepatic mRNA expression of SREBP-1 and fatty acid synthase (FAS), which could contribute to improved hepatic lipid metabolism [126]. Other recent findings suggest that phenolic BB extract improved hepatic lipid metabolism via pathways involving the bile acids receptors farnesoid X receptor (FXR) and Takeda G protein-coupled receptor 5 (TGR5) [33].

Moreover, BB phytochemicals elicit mitochondrial-targeted protective properties by scavenging ROS and acting as uncouplers of the OXPHOS, preserving mitochondrial function and protecting cells from apoptosis [144,145,146,147,148] (Figure 1). Likewise, a protective effect of BB anthocyanin-rich extract was found in mice hepatic mitochondria against acrylamide-induced mitochondrial oxidative stress by inhibition of ROS formation [148]. At the cellular level, C3G (one of the main components of BAE) reduced ROS formation, caspase-3 and -9 inactivation, and downregulated the pro-apoptotic Bax protein induced by hyperglycemia, preventing mitochondrial dysfunction through modulation of PI3K/AKT and JNK signaling pathways [145]. The combination of BB juice and probiotics (BJP) reduced NAFLD-induced mitochondrial ultrastructure damage and swelling and hepatic necrosis in parallel with improvements on respiratory function. Moreover, BJP attenuated mitochondrial oxidative stress through elevation of GSH and SOD levels while reducing ROS production in an animal model of NAFLD. The authors demonstrated that the modulation of SIRT1/PGC-1α pathway is a potential target of BJP against hepatic damage induced by NAFLD [146]. More recently, the activation of the AMPK/PGC-1α/SIRT3 signaling pathway was associated with beneficial effects of BB leaf polyphenols on hepatic mitochondrial function and oxidative defense [140]. Additionally, improvements on mitochondrial respiratory parameters were found in isolated hepatic mitochondria from non-obese diabetic Goto-Kakizaki (GK) rats after drinking bilberry leaves decoction for 4 weeks [149]. Even though a mechanistic explanation was not scrutinized in this study, quercetin present in bilberry leaves may partially explain the increased mitochondrial oxidative and phosphorylative activities in GK treated rats. In fact, this polyphenol has a positive impact on mitochondrial biogenesis related with an increased mRNA expression of PGC-1α and SIRT1 along with higher contents of mtDNA and cytochrome c [150]. Remarkably, recent data from our group revealed that long-term BB fruits intake in a juice form elicited an accentuated bioenergetic remodeling in isolated hepatic mitochondria along with a metabolic transcriptional adaptive response in healthy animals [130]. Further animal research also showed that the attenuation of hepatic steatosis and enhancement of lipolysis following exposure of hepatic cells to BB polyphenols can be promoted by modulation of autophagy [36].

In vivo animal studies have showed that BB reduced the aspartate aminotransferase (AST) and alanine aminotransferase (ALT) levels in both mouse models of liver injury but also in obese and insulin resistant animal models [32,151]. Conversely, despite changes in lipid peroxidation in the liver, the hepatic TGs accumulation and serum AST and ALT levels were not altered following the administration of whole blueberry powder or polyphenolic-rich extracts in diet-induced obesity and insulin resistant mice [152]. Interestingly, authors found that the improved metabolic outcomes were only exhibited in obese insulin resistant animals supplemented with BB polyphenol-rich fractions and no improvements were found in mice fed high-fat high-sucrose diet plus whole BB powder, suggesting that the metabolic benefits are a result of specific classes of polyphenols isolated from the whole fruit.

In humans, some interventional studies have demonstrated the ability of BB to lower hepatic transaminases (such as ALT and AST), which might suggest hepatoprotection [105,113]. Moreover, a double-blind RCT conducted in patients with NAFLD for 12 weeks demonstrated beneficial effects of purified anthocyanins derived from bilberry and black currant by improving insulin resistance [114].

**Table 2:** Blueberries hepatoprotective effects in (pre)diabetic animal models.

Animal Model	Source/Dose	Duration	Main Outcomes	Ref
HFD-fed male C57BL/6J mice	BB anthocyanin extract (200 mg/kg BW)	8 weeks	↓ BW, liver and adipose tissue weight ↓ Insulin levels ↓ HOMA-IR ↓ TGs and T-Chol ↓ Hepatic steatosis ↓ Serum ceramide and DAG levels ↓ Serum PP2A and Prkcz mRNA expression ↓ ALT and AST activities	[32]
Obese Zucker rats	Freeze-dried wild BB powder (8% of diet)	8 weeks	= BW ↓ Fasting plasma TAG and T-Chol ↓ Hepatic SREBP-1 and FAS mRNA expression = Hepatic PPAR-α and PPAR-γ mRNA expression	[126]
			↓ Fasting plasma TNF- α, IL-6, and CRP ↑ Plasma adiponectin ↓ Hepatic TNF- α, IL-6, CRP and NF-kB mRNA expression	[153]
HFD-fed Sprague Dawley rat	BB juice (15 g/kg) combined with probiotics	8 weeks	↓ Serum TGs, T-Chol, LDL-c and MDA ↓ Hepatic lipid accumulation ↑ Hepatic SIRT1and PPAR-α mRNA and protein expression ↓ Hepatic mRNA levels of SREBP-1c ↑ Serum SOD and GSH activities ↓ Serum ALT and AST activities	[151]
			↑ Hepatic mitochondrial swelling ↓ State 3 and 4 respiration rates ↑ RCR and ADP/O ratio ↑ Hepatic mitochondrial GSH and SOD mRNA and protein expression levels ↑ Hepatic protein and mRNA expression of SIRT1 ↑ Hepatic mRNA expression of PGC-1α ↓ Hepatic mitochondrial MDA levels and ROS activity	[146]
HFD-fed obese postmenopausal female C57BL/6J mice	BB powder (4% of diet)	12 weeks	= BW and fat mass ↑ Glucose tolerance ↓ Hepatic steatosis ↑ Hepatic FAO and lipid handling (↑ LCHAD, and FAT/CD36 mRNA expression) ↑ Fecal SCFAs levels	[154]
HFD-fed C57BL/6J mice	BB polyphenol extract (200 mg/kg body weight (bw)/day)	12 weeks	↓ BW ↓ Serum LDL -c levels ↑ Serum HDL-c levels ↓ Hepatic TGS and T-Chol ↓ Hepatic PPAR-γ, FAS, and SREBP-1 mRNA expression ↑ Hepatic CPT1 and PPAR-α mRNA expression ↑ AMPK phosphorylation	[34]
HFD-fed male C57BL/6J mice	BB anthocyanins (200 mg/kg)	12 weeks	↓ BW ↓ Serum and hepatic lipid and MDA levels ↑ Hepatic SOD and GPx activities ↓ TNF-α, IL-6 and NF-kB mRNA expression ↑ Fecal SCFAS Hepatic glycerophospholipids and glutathione metabolism and insulin-signaling pathways modulation	[155]
Diabetic male KK-Ay mice	BB anthocyanin extract (10 g/Kg diet)	4 weeks	= BW and ↓ liver weight ↓ Serum glucose levels ↑ Insulin sensitivity ↓ Serum TGs and TC-Chol ↓ HGP ↑ Hepatic AMPK phosphorylation ↓ Hepatic lipid content ↓ Hepatic gluconeogenesis (↓ PEPCK and G6Pase mRNA expression) ↑ FAO (↑ PPAR-α, ACO and CPT1 mRNA expression)	[136]
HFD-fed C57BL/6J mice	Fermented BB juice	17 weeks	↑ BW gain ↑ Insulin sensitivity and glucose homeostasis ↓ Fasting serum glucose ↓ Serum LDL-c ↓ Serum TNF-α and ↑ IL-10 ↓ Hepatic fat accumulation ↑ Hepatic mRNA expression of IR and IRS ↓ Hepatic SCD1, SREBP1c and FAS expression ↑ mRNA and protein expression of GLUT-1; GCK, LDL-receptor and PPAR-α ↓ mRNA and protein expression of PPAR-γ ↑ Akt phosphorylation	[156]
GK rats	Decoctions of *Vaccinium myrtillus* L. (bilberry) leaves	4 weeks	= BW ↑ Glucose tolerance ↓ Occasional glycaemia ↑ Mitochondrial oxidative and phosphorylative activities ↑ State 3 respiration, FCCP-uncoupled respiratory and RCR activities	[149]

ACO, acyl-CoA oxidase; Akt, protein kinase B; ALT, alanine aminotransferase; AMPK, 5′ adenosine monophosphate-activated protein kinase; AST, aspartate aminotransferase; BW, body weight; CPT1, carnitinepalmitoyltransferase-1A; DAG, diacylglycerol; FAO, fatty acid oxidation; FAS, fatty acid synthase; FCCP, carbonyl cyanide p-(tri-fluromethoxy)phenyl-hydrazone; G6Pase, glucose 6-phosphatase; GCK, Glucokinase; GLUT-1, glucose transporter 1; GPx, glutathione peroxidase; GK, Goto-Kakizaki; HGP, hepatic glucose production; HFD, high-fat diet; HOMA-IR, homeostatic model assessment for insulin resistance; IL, interleukin; LCHAD, long-chain hydroxyacyl-CoA dehydrogenase; LDL-c, low-density lipoprotein-cholesterol; MDA, malondialdehyde; PEPCK, phosphoenolpyruvate carboxykinase; RCR, respiratory control ratio; SCD1, stearoyl-CoA desaturase-1; SCFAS, short-chain fatty acids; SOD, superoxide dismutase; SREBP-1, sterol regulatory element-binding protein-1; TNF-α, tumor necrosis factor-α; T-Chol, total-cholesterol; ↑, increased; ↓, decreased; =, unchanged.

### 3.5. Prebiotic-like Effects of BB

Given that BB phytochemical composition comprises several bioactive compounds including high molecular weight PP and easily fermentable dietary fibers that are not well absorbed in the small intestine and reach the large intestine, such bioactive components are available to interact with GM [157]. Indeed, prebiotic-like properties and/or antimicrobial effects against pathogenic intestinal bacteria of BB and their bioactive compounds have been reported in in vitro assays [116,158,159], in animal models [152,160] and in human intervention studies [115,161], as outlined in Figure 1. An in vitro study reported distinct positive correlations between different PP contents and bacterial species; in fact, while PP from BB bagasse powder was correlated with Actinobacteria (*Bifidobacterium* and *Colllisella*) and *Akkermansia*, the fiber content was associated with *Faecalibacterium* and *Bifidobacterium* [159]. Moreover, the presence of anthocyanins and fibers in BB pomace promoted the growth of *Lactobacillus* and *Ruminococcaceae* genus and was negatively associated with *Streptococcus*. The addition of a BB extract to mixed human fecal bacterial populations enhanced the population size of Lactobacilli and Bifidobacteria [160].

Growing evidence hint that the positive effects of BB in improving glucose metabolism and energy balance are closely linked with their ability to modulate the composition and/or function of GM [34,152,157,162]. Table 3 summarizes the most relevant studies performed in animal models. A recent study reported that upon intestinal absorption BB polyphenols improved metabolic health in diet-induced obese mice via modulation of GM rather than through systemic effects [152]. It was also observed that germ-free mice receiving fecal microbiota transplantation (FMT) from mice fed BB polyphenolic fractions (specifically enriched with proanthocyanins or anthocyanidins) were protected from diet-induced obesity and presented an improved insulin sensitivity and glucose homeostasis when compared to mice receiving GM from HFHS fed mice. Furthermore, authors postulated that the beneficial effects of FMT could be the result of a decreased proportion of BCAA in the feces of mice fed whole BB or just the polyphenols [152]. Moreover, positive effects on insulin sensitivity and glycemia were found in the mouse model of HFD-induced obesity upon BB juice supplementation for 17 weeks, with or without fermentation [156]; the authors hypothesized that this protection was closely related with BB ability to stimulate SCFAs production and maintain GM homeostasis [156].

Among health commensal bacteria, *Akkermansia muciniphila* (AM) has been positively correlated with enhanced mucosal barrier function and improved metabolic phenotype in obesity ad T2DM [116,164,165,166]. Notably, BB interventions enhanced AM levels in animal models of metabolic syndrome [162,167]. Indeed, whilst favouring the growth of healthier bacteria, BB constituents seem to display the ability to restore gastrointestinal integrity by: (i) enhancing colonic mucus thickness [162]; (ii) increasing the expression of tight junctions proteins [168]; (iii) overexpressing MUC-2 mRNA, the primary glycoprotein of the gastrointestinal mucus layer [127]; (iv) reducing endotoxemia and intestinal inflammation [33,127] and (v) fortifying the mucosal immunity [167].

Interestingly, BB polymeric proanthocyanidins have been recently shown capacity to restore the colonic mucus layer, modulate GM and attenuate glucose tolerance in obese mice, in contrast to the anthocyanin fraction from BB [162]. However, other subclasses of polyphenols, secondary metabolites and fibers also present in BB may potentially account for these prebiotic effects counteracting the metabolic disorders [35,116,169]. Notwithstanding, ambiguous studies and conflicting results still prevail, which could possibly be explained by several factors such as distinct duration and mode of administration of BB interventions, differences in the BB phytochemical composition (i.e., the type and structure of polyphenolic constituents), as well as differences in the dietary regimens. Noteworthy, the inclusion of bilberries in the high-fat setting prompted an increase in SCFA contents accompanied by the normalized α-diversity of GM, enhanced the abundance of Bacteroidetes, *Bifidobacterium* and butyrate-producing bacteria, which collectively contributed to thwart pre-obesity events like hepatic lipid accumulation [163].

The impact of BB on GM has been also evaluated in human interventional studies [161]. Vendrame et al. [115] showed that 6 weeks consumption of a BB powder drink rich in anthocyanins significantly enhanced the abundance of Bifidobacteria in the cecum from healthy volunteers, whereas no differences were observed for *Bacteroides* spp., *Prevotella* spp., *Enterococcus* spp., and *Clostridium coccoides*. A pilot RCT was carried out in overweight and obese subjects to evaluate the effects of a gastrointestinal microbiome modulator (GIMM), composed of inulin, β-glucan and anthocyanins and polyphenols from BB, on metabolic parameters, fecal markers of GM, and satiety [170]. Thirty overweight or obese participants consumed the GIMM or placebo, daily, for 4 weeks. Improved glucose tolerance restored satiety hormones (increased PYY and decreased ghrelin) and fecal SCFAs contents were observed in the GIMM-treated group when compared to the placebo; however, no significant changes were found between groups regarding insulin sensitivity, fecal markers of GM, plasma satiety hormones or serum lipid concentrations [170]. The prebiotic effects of BB reported in the preclinical ground need further validation in the clinical setting, namely in the (pre)diabetic state, as the literature is particularly scarce in human interventions in this condition. There is one ongoing clinical trial (NCT03266055) exploring the prebiotic effects of BB in subjects with metabolic syndrome. The results from this and other trials further conducted will be decisive to achieve more robust conclusions regarding the use of BB to prevent dysbiosis in prediabetes.

## 4. Concludings

Herein, we provide a review of the current knowledge in this field by revisiting preclinical data obtained from in vitro assays and animal models together with studies from human subjects, to disclose the mechanisms by which BB may be a valuable approach in prediabetes (Figure 1). Through improving glycemic control, reducing oxidative stress and inflammation, regulating TG’s accumulation and trafficking, along with ameliorating GM dysbiosis, this multi-modal functional fruit positively impacts a diversity of pathophysiological mechanisms that occur as early as prediabetes and for which pharmacological interventions (either oral antidiabetics or weight-loss drugs) are not approved in practical guidelines [17] or are only considered in cases at high risk [171]. In this sense, BB have been shown to modulate hepatic and intestinal molecular targets of currently approved antidiabetics [α-glucosidase inhibitors (e.g., acarbose), DPP-4 inhibitors (e.g., sitagliptin), PT1B inhibitors (rosiglitazone)] [98,172,173], whose benefits often surpass the glucocentric view of diabetes management [174,175]. As a matter of fact, BB benefits also goes beyond the improvement of glycemic control as they positively modulate a panoply of metabolic functions that ensues not only in the pancreas but also in the gut and the liver, two pathophysiologic paths that critically determine (pre)diabetes progression. Collectively, it is outlined how blueberries may provide a fruitful source of phytochemicals able to prevent (pre)diabetes progression.

As final critical analysis, two serious limitations narrow our current understanding of BB health benefits on (pre)diabetes. Firstly, many in vivo and in vitro studies were conducted with BB-derived PP in supraphysiological doses, unlikely to be achieved upon human daily consumption. Secondly, most in vitro studies use undigested dietary BB-derived polyphenols and miss the corresponding in vivo metabolites/conjugates. Collectively, these data have a low predictive value. Finally, RCTs greatly diverge on BB supplementation regimens. Future research should focus on the qualitative and quantitative evaluation of BB polyphenols in both systemic circulation and target tissues. Moreover, longer-term RCTs with standardized interventions (e.g., doses employed, duration) and readouts will hopefully bring stronger evidence to guide a more effective use of this functional fruit with remarkable antioxidant activity in the early stage of diabetes.

## Figures and Tables

**Table 1:** Blueberries effects on biomarkers relevant to T2DM and associated metabolic disorders—clinical evidence.

Subjects	Source/Dose	Duration	Main Metabolic Outcome	Ref
Participants with metabolic syndrome	Freeze-dried BB (26 g of powder, equivalent to 150 g of fresh BB)	6 months	= Insulin Resistance = HOMA-IR, HbA1c and QUICKI = Blood pressure ↑ HDL and apoA-1 ↑ Vascular functions ↓ Systemic arterial stiffness ↑ Artery endothelial function	[107]
Obese subjects with metabolic syndrome (pilot study)	Meals enriched with BB	2–4 h	↓ Urinary ketone body levels ↓ Urinary succinate levels ↓ Dimethylamine and trimethylamine ↑ TGF-β mRNA expression from blood mononuclear cells ↓ IL-6 mRNA expression from blood mononuclear cells	[108]
Obese nondiabetic and insulin resistant subjects	Freeze-dried BB (total 45 g powder; ≈ 2 cups of fresh BB)	6 weeks	↑ Insulin sensitivity ↓ Superoxide and total ROS in whole blood and monocytes ↓ Monocytes mRNA expression of TNF-α, TLR4 and IL-6	[109]
Subjects with metabolic syndrome	Freeze-dried BB beverage (50 g, ≈350 g fresh BB)	8 weeks	= Glucose levels and lipid profiles ↓ Blood pressure ↓ Oxidized LDL ↓ Oxidative stress markers (MDA and 4-HNE). = Plasma CRP, IL-6 MPO, adhesion molecules and adiponectin	[110]
T2DM patients	Purified anthocyanins (320 mg/day ≈ 100 gr fresh BB and blackcurrants)	24 weeks	↓ LDL-c, TGs, apo-B and apoC-III ↑ HDL ↓ Fasting glucose and HOMA-IR ↑ Adiponectin and β-hydroxybutyrate ↓ Serum IL-6 and TNF-α ↑ Antioxidant capacity	[111]
Subjects with metabolic syndrome	Lyophilized BB extract (BlueKing^®^, 2 chewable tablets/day 1200 mg of anthocyanins)	6 months	= Glucose and insulin levels = T-Chol and TGs contents ↓ DNA damage ↓ Vascular endothelium and cell membranes damage ↓ Urinary biomarkers of oxidative stress (8(OH)dG and F2-isoprostanes) ↓ Urinary uric acid ↓ hs-CRP	[112]
T2DM subjects	Single capsule of Mirtoselect^®^ standardized BB extract (0.47 g, 36 % anthocyanins; 50 g of fresh BB	1 day and 2-week washout period	↓ AUC of GTT ↓ Postprandial glucose levels ↓ Plasma insulin levels = Plasma GIP, GLP-1, glucagon and amylin = Plasma MCP-1 inflammatory adipokine and oxidative stress markers	[104]
Men with T2DM	Freeze-dried BB (22 g, equivalent to ∼1 cup fresh per day)	8 weeks	= BW and blood pressure = Fasting plasma glucose and insulin = LDL-c, HDL and T-Chol = CRP ↓ HbA1c and fructosamine ↓ TGs ↓ Hepatic enzymes ALT and AST	[105]
Overweight and obese subjects	BB (50 g serving)	12 weeks	↓ BW, body fat and BMI ↓ LDL-c, and T-Chol ↓ HbA1c, insulin and insulin resistance ↓ Hepatic enzyme AST	[113]
Adults at risk for T2DM	100% wild BB juice (240 mL/day)	7 days	= TGs, glucose and insulin levels = Insulin sensitivity, inflammatory markers, adhesion molecules, oxidative stress, endothelial function, or blood pressure. ↓ Systolic blood pressure ↑ Serum nitrates and nitrites levels	[30]
Obese, nondiabetic, and insulin-resistant subjects	BB powder (45 g, equivalent to ~2 cups of fresh BB)	6 weeks	= BW ↑ Insulin sensitivity = Adiposity, energy intake = Serum TNF-α, MCP-1 and hs-CRP levels	[31]
Patients with non-alcoholic fatty liver disease	Purified anthocyanin (320 mg/d) derived from bilberry and black currant	12 weeks	↓ Glucose intolerance ↓ Plasma TGs ↓ ALT ↓ CK-18 M30, a predictor of NAFLD progression ↓ MPO	[114]
Healthy male individuals	Wild BB powder (25 g in 250 mL of water)	6 weeks	↑ Bifidobacterium spp. ↑ *Lactobacillus acidophillus* = *Bacteroides* spp., *Prevotella* spp., *Enterococcus* spp., and *Clostridium coccoides.*	[115]
Healthy old (aged 65–77 years) women	Freeze-dried BBs (38 g/day)	6 weeks	↑ α-microbiota diversity ↑ Healthy microbiota: *Faecalibacterium-prauznitzii*, *B. Intestinihominis*, *Ruminococcus bromii*, *Eubacteriu halli*. *Anaerostipes hadrus* and *Butyrisimonas virosa*	[116]

4-HNE, 4-Hydroxynonenal; 8-OHdG, 8-hydroxy-2′-deoxyguanosine; AUC, area under the curve; CK-18, cytokeratin 18; GIP, gastric inhibitory polypeptide; GTT, glucose tolerance test; HbA1c, glycated hemoglobin; HDL, high-density lipoprotein; hs-CRP, high-sensitivity C-reactive protein; MCP-1, monocyte chemoattractant protein 1; MPO, myeloperoxidase; QUICKI, quantitative insulin sensitivity check index; TGF-β, transforming growth factor beta; ↑, increased; ↓, decreased; =, unchanged.

**Table 3:** Blueberries gut modulatory and prebiotic effects in T2DM or metabolic syndrome animal models.

Animal Model	Source/Dose	Duration	Main Outcomes	Ref
HFD-fed C57BL/6J mice	BB polyphenol extract (200 mg/kg)	12 weeks	↓ *Prevotella* and *Clostridium* ↓ *Lactobacillus spp.* and *Coprobacillus* ↑ *Proteobacteria* and *Helicobacter* ↑ *Bifidobacterium*	[34]
HFHS diet-fed male C57BL/6J mice	BB polyphenol extract (200 mg/kg)	8 weeks	↑ Glucose tolerance ↑ Colonic mucus thickness ↑ *Ruminococcaceae* and *Peptostreptococcaceae* ↓ *Bacteroidetes* phylum = *Akkermansia muciniphila* ↑ *Adlercreutzia equolifaciens*	[162]
	BB oligomeric proanthocyanidins fraction (53 mg/kg)	8 weeks	↑ Glucose tolerance ↑ Colonic mucus thickness ↑ *Akkermansia muciniphila* ↑ *Adlercreutzia equolifaciens* ↓ *Ruminococcaceae* and *Peptostreptococcaceae*	
HFD-fed male C57BL/6J mice and diabetic male C57BL/KsJ db/db mice	BB phenolic extract (5 g/L in drinking water)	14 weeks	↓ BW gain and adiposity ↑ Glucose tolerance and insulin sensitivity ↓ Plasma LPS, IL-6, TNF-α contents ↓ Hepatic steatosis ↓ Plasmatic and hepatic TGs ↓ Serum LDH, ALT and AST ↑ Intestinal MUC-2 levels ↑ Intestinal occludin and ZO-1 mRNA expression ↓ Intestinal and hepatic inflammation (TLR4, IL-6, TNF-α) ↑ *Bifidobacteria* and *Akkermansia* ↓ *Desulfovibrio* and *Bilophila genera* ↓ Plasma BA pool size ↑ mRNA and protein expression of FXR and TGR5 ↓ mRNA expression levels of SREBP-1c and ChREBP and downstream genes	[33]
HFD-fed male Sprague Dawley rats	Dietary fiber from BB (7% of diet)	8 weeks	↓ BW gain ↓ Serum T-Chol levels ↓ Hepatic steratosis ↓ Hepatic mRNA expression of SCD1 = Hepatic mRNA expression of genes involved in lipogenesis and β-oxidation ↑ mRNA expression of Ucp-1 ↑ Fecal and serum total SCFAs ↑ Butyrate-producing bacteria ↑ Actinobacteria and Proteobacteria ↓ Firmicutes and ↑ Bacteroidetes = *Bifidobacterium*	[163]
HFHS diet-fed male C57BL/6 mice	Freeze dried BB powder (4% of diet, 160 mg/day)	12 weeks	= BW and liver weight = HOMA-IR = Glucose homeostasis = Hepatic TGs, T-Chol = Plasma ALT and AST ↓ Fecal BCFA isobutyric and isovaleric acid ↓ *Ruminococcus*, *Lachnospiraceae* and *Blautia*	[152]
	BB proanthocyanidin extract (1 mg/day)	12 weeks	↓ BW and epididymal adipose tissue weight ↓ Plasma insulin and C-peptide levels ↓ HOMA-IR ↑ Insulin sensitivity = Hepatic TGs, T-Chol = Plasma ALT and AST ↓ BCFA isobutyric and isovaleric acid ↑ *Muribaculum inestinale* abundance ↑ Oxidative phosphorylation pathway ↑ Homeostasis and insulin sensitivity after transferred by FMT	
HFD-fed Wistar rats	Freeze-dried BB powder (10% of diet	8 weeks	= BW ↑ Insulin sensitivity ↓ Serum LBP levels ↓ Hepatic p-IRS1 to IRS1 ratio ↓ Hepatic MDA concentration ↑ Ileal mRNA expression of Muc-2 ↓ Ileal mRNA expression of TNF-α ↑ Goblet cell number per crypt ↑ Gammaproteobacteria (*Actinobacillus* and *Aggregatibacter*) ↑ *Lactobaccilus* ↓ Firmicutes and Bacteroidetes ↑ Serum SCFAs (acetate and propionate) ↑ Ileal mRNA expression of GLP-1	[127]
HFD-fed C57BL/6J mice	Fermented BB juice and fresh BB juice	17 weeks	↑ Cecum SCFAs (acetate and valerate) ↑ Bacteroidetes (*Barnesiella*) ↓ Firmicutes ↑ *Akkermansia, Bifidobacterium, and Lactobacillus* ↓ Obese-type gut microbiota (*Oscillibacter* and *Alistipes*)	[156]

BA, bile acids; BCFA, branched chain fatty acids; FMT, fecal microbiota transplant; FXR, farnesoid X receptor; GLP-1, glucagon-like peptide-1; HFD, high-fat diet; HFHS, high-fat high sucrose; IRS-1, insulin receptor substrate-1; LBP, lipopolysaccharide-binding protein; LDH, lactate dehydrogenase; LPS, lipopolysaccharides; Muc-2, mucin 2; SCFAs, short chain fatty acids; TGR5, G-protein-coupled bile acid receptor; TLR4, Toll-like receptor 4; Ucp-1, uncoupling protein-1; ZO-1, zonula occludens-1; ↑, increased; ↓, decreased; =, unchanged.
